# Sporotrichosis in the earlobe caused by placing an earing

**DOI:** 10.1016/j.bjid.2024.104464

**Published:** 2025-01-02

**Authors:** Evelyn Zacarias, Talita Alves, Claudilson Bastos, Paulo Athanazio, Sergio Arruda

**Affiliations:** a Instituto Gonçalo Moniz – FIOCRUZ, Salvador, BA, Brazil; b Instituto Couto Maia ‒ SESAB, Salvador, BA, Brazil; c Laboratório IMAGEPAT, Salvador, BA, Brazil; d Universidade Estadual da Bahia (UNEB), Salvador, BA, Brazil

**Keywords:** Sporotrichosis, Earlobe, Atypical form

## Abstract

A 22-year-old woman presented with an ulcer on her right earlobe 2 months ago, with inflammation and enlarged ipsilateral lymph nodes in her neck. She was treated with antibiotics without success and then was referred to an infectious disease specialist. She has a cat at home with sporotrichosis, but without direct contact with the lesion, she did not remember any scratching by the cat. She also mentioned wearing a semi-jewel earring. This is a rare and unusual case of sporotrichosis in the earlobe, probably caused by wearing an earring contaminated by the cat's fungus that was present in the home environment. The delay in diagnosis and treatment led to the worsening of the injury and loss of the earlobe.

## Introduction

Sporotrichosis is a subacute or chronic mycosis caused in most cases by traumatic inoculation of the dimorphic fungus *Sporothrix schenckii* and other species.^1^ It is one the most common subcutaneous mycosis in Latin America, with worldwide distribution. The disease has recently reached epidemic proportions in some regions of Brazil, such as Rio de Janeiro, where the number of cases of zoonotic transmission by infected cats has increased significantly.^2^ The traditional form of transmission is by traumatic inoculation of the fungus into the skin, through contact with contaminated plants, organic substrates, or infected animals.^3^ The most common clinical presentation is lymphocutaneous. The disease has been classified into three different clinical forms: cutaneous lymphatic, fixed, and disseminated.^3^ In adults, the most common location is the upper limbs, while in children it tends to occur more commonly on the face.^3^ Although the traditional cutaneous-lymphatic form represents most cases of sporotrichosis, the increased incidence of the disease in Brazil has led to an increase in the incidence of atypical and severe clinical forms. We describe a case of sporotrichosis in a unique location (the ear pinna) with an unusual transmission mode. The delay in the diagnosis and treatment resulted in higher morbidity, unsightly scarring, and loss of the ear lobe.

## Case report

**Fig. 1:**
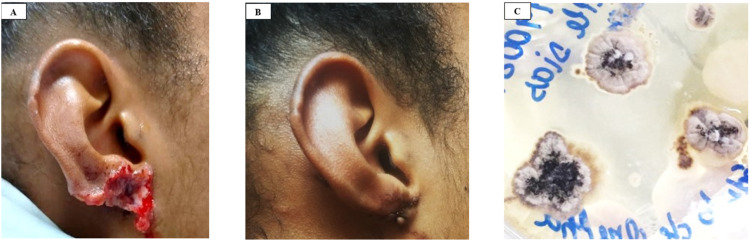
Ear lobe lesions pre (A) and post (B) treatment. In C shows black colonies grown from tissue fungi cultivation.

A 22-year-old patient reported an ulcerated skin lesion in the right ear lobe with an approximate duration of 2 months, evolving with local inflammatory signs and ipsilateral cervical lymphadenopathy (Fig. 1). She was seen by a dermatologist and the antibiotic Ceclor BD® (cephalosporin 500 mg) was prescribed for 10 days, with no improvement. When she returned to the dermatologist, she was referred for evaluation by an infectious disease specialist.

The patient reports the presence of a sick cat with sporotrichosis in her home but without a report of biting, scratching, or licking at the lesion site. She refers to wearing an earring, the composition being a semi-jewel. The treatment for this patient was Terbinafine 250 mg/day for seventeen days, and after this Itraconazole 400 mg/day for two months, the patient was cured of the infection and presented unsightly scarring of the earlobe.

## Discussion

In the literature, there is only one similar report described in Rio de Janeiro, Brazil in a patient who also her earlobe was also infected by *Sporotrix* sp. when putting on an earring.^4^ In most cases of cutaneous sporotrichosis, the fungus is inoculated into human skin through scratches from the sick animal present at home. In this case report the patient does not mention that the sick cat scratched her ear, suggesting that the fungus was inoculated by the act of putting on the earring, indicating the presence of the fungus in the environment including the jewelry, an unusual form of infection. This case demonstrates the importance of reporting the presence of a sick animal in the owner's home to ensure an early clinical diagnosis to prevent the development of a deforming injury.

**Fig. 2:**
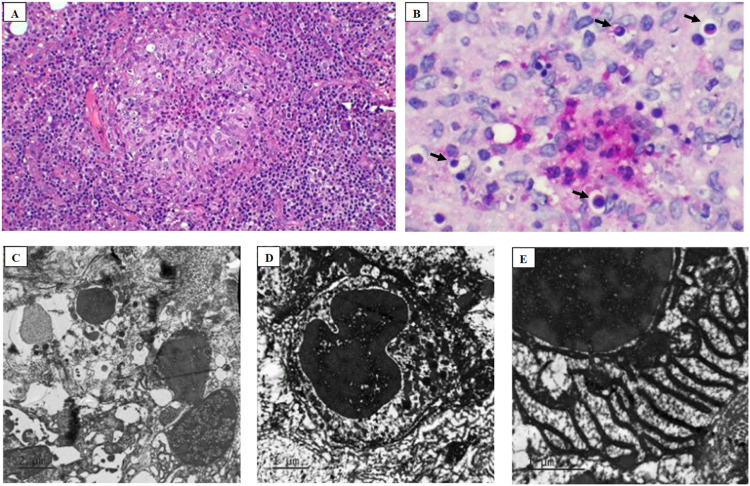
Histopathology analysis in A with Hematoxylin Eosin (4 ×) stained biopsy, showing a granuloma with cellular debris in the center, and B PAS (20 ×) stained showing fungi (arrows) elements identified as *Sporotrix* bodies. Photomicrography images of transmission electron microscopy in C, D, and E shows the ultrastructure of the *Sporotrix*.

The main diagnosis of human sporotrichosis is the presence of *Sporothrix* fungi in the lesion, which can be identified from a biopsy sent for histopathological analysis and isolation in culture. In this case, both searches were carried out. Cultivation of the fungus from skin biopsy identified the growth of a fungus with dark colonies diagnosed as *Sporothrix* spp. (Fig. 1C). In histopathology with Hematoxylin-Eosin (HE) and Periodic Acid-Schiff (PAS) staining, it was possible to identify an intense inflammatory infiltrate with fungi in the form of yeast with a round shape and thick capsule (Fig. 2A and B). Additionally, transmission electron microscopy of the lesion biopsy was performed, identifying the ultrastructure of the fungus (Fig. 2C–E).

## Conclusion

The growing number of new cases of sporotrichosis in Brazil allows the disease to be transmitted in unusual ways, which can make rapid diagnostic elucidation difficult. Late diagnosis can result in large tissue destruction and scars after treatment that create discomfort for the patient. Thus, given the Brazilian epidemic of sporotrichosis, the importance of associating clinical history and the presence of sick animals in the home is verified even in unusual and small lesions with an ulcerative pattern associated with lymphadenopathy.

## Ethics statement

The study was approved by the Ethical Committee from Hospital Couto Maia (CAE 77810124.6.0000.0046).

## Funding

10.13039/501100006507Fundação Oswaldo Cruz.

## Conflicts of interest

The authors declare no conflicts of interest.
